# Disease modeling for public health: added value, challenges, and institutional constraints

**DOI:** 10.1057/s41271-019-00206-0

**Published:** 2019-11-28

**Authors:** Mirjam Kretzschmar

**Affiliations:** 1 Center for Infectious Disease Control, National Institute of Public Health and the Environment (RIVM), Bilthoven, The Netherlands; 2 Julius Center for Health Sciences & Primary Care, University Medical Center Utrecht, Utrecht University, Utrecht, The Netherlands

**Keywords:** Mathematical model, Public health, Policy support, Infrastructure

## Abstract

Public health policymakers face increasingly complex questions and decisions and need to deal with an increasing quantity of data and information. For policy advisors to make use of scientific evidence and to assess available intervention options effectively and therefore indirectly for those deciding on and implementing public health policies, mathematical modeling has proven to be a useful tool. In some areas, the use of mathematical modeling for public health policy support has become standard practice at various levels of decision-making. To make use of this tool effectively within public health organizations, it is necessary to provide good infrastructure and ensure close collaboration between modelers and policymakers. Based on experience from a national public health institute, we discuss the strategic requirements for good modeling practice for public health. For modeling to be of maximal value for a public health institute, the organization and budgeting of mathematical modeling should be transparent, and a long-term strategy for how to position and develop mathematical modeling should be in place.

## Mathematical modeling for policy support

Public health policymakers confront increasingly complex questions and decisions and need to deal with increasing amounts of data and information. Public health institutes form a link between scientific research and public health policymaking and practice, and in this role they provide guidance and advice to public health policymakers. To do so, they need to make use of scientific evidence and to assess available intervention options effectively. For researchers and policy advisors who compile and evaluate scientific evidence for health interventions, mathematical modeling has proven to be a useful tool. Developing a mathematical model helps to synthesize information from different sources into a consistent framework that allows an integrated analysis of complex problems [1, 2]. Researchers in public health, who provide advice to policymakers, often use mathematical models to simulate the impact of various interventions or public health strategies, and to provide quantitative predictions of how interventions might affect population health in the future.

In some areas, the use of mathematical modeling for public health policy support has become standard practice at various levels of decision-making [3–5]. In the field of infectious disease control, mathematical modeling has a long history and has become an important tool in decision-making for public health in the last two decades. During the influenza pandemic of 2009, mathematical modeling helped the World Health Organization (WHO) outbreak response team and decision-makers in national outbreak response units with interpretation of outbreak data during the early phase of the epidemic [3]. Results from modeling studies also supported decisions about vaccination strategies during later stages of the outbreak by providing estimates of the basic reproduction number and evaluating how timing and targeting of vaccination to different population groups might impact the epidemic peak and duration [6–8]. More recently, during the large Ebola outbreak in West Africa, mathematical modelers estimated key parameters for outbreak control such as the impact of case isolation, contact-tracing with quarantine, and sanitary funeral practices on the numbers of new infections [9, 10]. When a vaccine against Ebola became available, mathematical modeling helped researchers and outbreak responders to design ring vaccination trials that could lead to successful testing of the vaccine despite a decreasing exposure risk during the declining epidemic phase [11, 12]. This experience has led the WHO to publish a guidance document on the design of vaccine efficacy trials during public health emergencies [13]. In the field of HIV prevention, modeling work by Granich and colleagues [14] has paved the way for UNAIDS to introduce the test-and-treat strategy with the long-term goal of elimination of HIV [15]. In the area of chronic diseases, models are used to generate projections of population health given demographic changes, distributions, and trends of risk factors in a population and possible effects of intervention programs [16–19].

Conceptual ideas and quantitative results from mathematical models are at the core of many reports and documents produced at public health institutes containing advice for policymakers. Therefore, modeling—often in combination with health economic assessments—potentially has great influence on policy decisions. Prominent examples include policy decisions concerning national immunization programs, where health authorities routinely perform or commission cost-effectiveness analyses before introducing new vaccines. The analysts usually base their work on ‘scenario analysis’ (i.e., a comparison of various possible intervention strategies in a systematic way) using ‘dynamic transmission models’ [20, 21]. The latter describe transmission between susceptible and infected individuals as a mechanistic process and are able to account for non-linear effects such as herd immunity.

Nevertheless, the contribution of modeling to generating the quantitative basis for public health information and decisions is often not visible and remains underrated by policymakers who base their decisions on advice produced by public health research and institutions. Even within public health institutes themselves, the importance of having broad and stable expertise in mathematical modeling is often undervalued. This creates a danger of insufficient investment for continuity and quality of modeling expertise. Responsible public health managers and communication officers, but also mathematical modelers themselves, need to make more effort to communicate with policymakers and public health professionals about the importance of models for policy analysis [22].

At present, several public health institutes around the world make use of mathematical modeling for policy advice. In the United Kingdom, the mathematical modeling unit of Public Health England works closely with the ministry of health. The Institute Pasteur in France, Robert Koch-Institute in Germany, and the National Institute of Public Health and the Environment (RIVM) in The Netherlands maintain modeling groups in their organizations. In North America, the United States (US) Centers for Disease Control and Prevention (CDC) and the Public Health Agency of Canada support internal modeling groups or collaborate with modelers in academia.

At the author’s organization, the RIVM, a dedicated group of senior scientists and policy advisors discussed the infrastructure and positioning of mathematical modeling with the aim of consolidating the existing expertise and developing a long-term strategy [23]. Here, we summarize uses of mathematical modeling, organization within the institute, and future challenges for disease modeling for public health.

## Areas of application and challenges

**Fig. 1:**
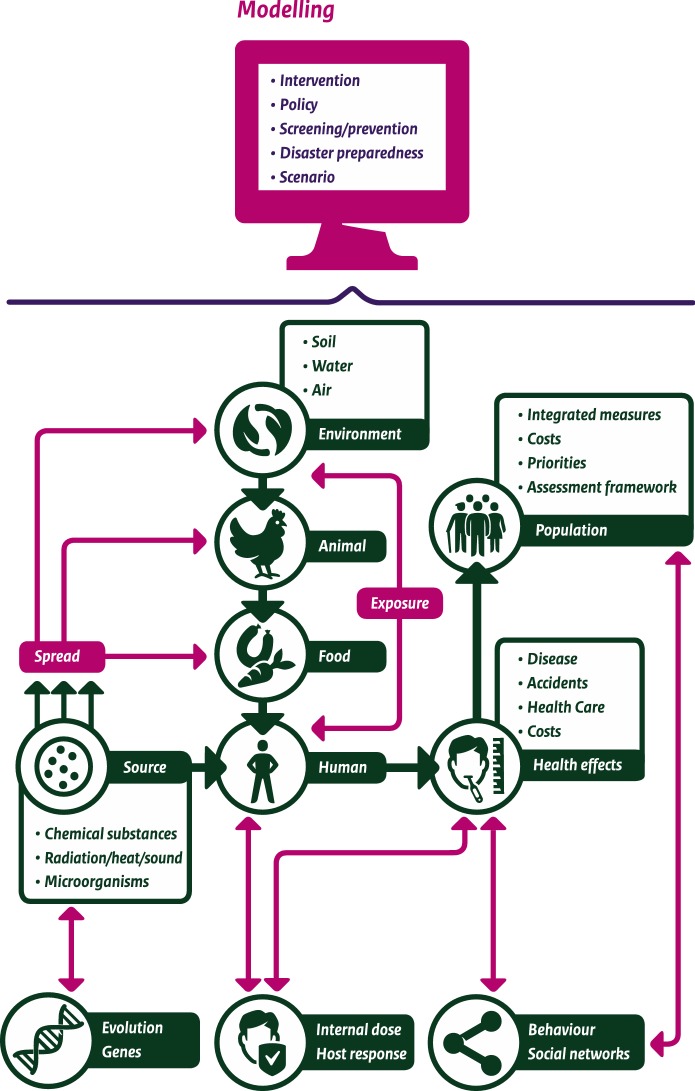
Applications of mathematical modeling

Mathematical modeling—broadly interpreted as using mathematical tools to conceptualize, formulate rigorously, and qualitatively and quantitatively analyze a problem at hand—permeates a large proportion of all research and policy advice produced at our institute, the National Institute for Public Health and the Environment (RIVM). The RIVM has a central role in infectious disease control and national prevention and population screening programs in the Netherlands and conducts independent (scientific) research in the field of public health, health services, environmental safety, and security. In this role, the RIVM produces numerous reports and publications on all aspects of public health, nutrition and food, health care, disaster management, nature, and the environment each year. Besides the ‘classical’ areas of application of mathematical modeling in infectious and chronic diseases, and to assess health effects of air pollution, we use models in the environmental sector for predicting the transport of substances through water and air [24], assessing the risks of exposure to toxic substances, and impact of radiation on health. Furthermore, there is a large area of application of microbial risk modeling [25] for establishing risk assessment for food safety or, more broadly, multi-criteria risk analysis including risk ranking of emerging infectious diseases [26]. In the health field as well as the environmental field, we use burden of disease measures, leading to the necessity of developing underlying models for exposure and disease progression [27, 28]. In public health, we use estimates of disease burden in terms of disability adjusted life years (DALYs) to compare the impact of various diseases on population health and to provide guidance for policymakers on how resources should be used effectively to achieve health gains in the population [29] (Fig. 1).

We use mathematical models on various levels of (biological) organization, namely within host modeling (including dose response models [30], models for development of cancer following radiation [31], models for immune response after vaccination [32]), population-level modeling (epidemiological models and health economic models), and complex system modeling [33, 34]. The last includes models used for strategic aims, such as describing interactions of stakeholder networks, modeling of health care systems (for example, waiting lists [35]), or models to help with complex decision-making.

Mathematical modelers in public health institutes are facing various types of challenges including practical ones of limited budgets and lack of infrastructure (see next section) and new scientific challenges posed by developments in other fields of science. One challenge of the latter type is the increased public health attention for risk perception and behavioral aspects of health and interventions. This has sparked collaboration between social scientists and modelers endeavoring to incorporate these factors into disease models [36]. Another—perhaps the most important—challenge of the last decade was the increasing availability of data from genome sequencing and other ‘omics’ data, or more generally big data sets [37].

Increasing availability and use of genomic data for diagnostic and surveillance purposes has revolutionized outbreak investigation, risk assessment, and epidemiological research. We need to develop new mathematical modeling tools to link genomic and other epidemiological data to create consistent frameworks. An entire new branch of modeling is developing; it can be subsumed under the term “phylodynamics” coined by Grenfell and colleagues [38]. A public health relevant application of such models is prediction of future strain composition of seasonal influenza using evolutionary models and genetic sequence information [39].

These developments in modeling methodology pose not only technical problems of availability of the required hardware and software, but also the necessity to develop knowledge among colleagues within the institute for using this type of data for public health. For mathematical modeling, it means that scientific computing is becoming increasingly important, which requires a further professionalization of computing. Where in the past smaller models could be developed and managed on desk top computers by modelers themselves, it is now becoming increasingly necessary to use high-performance computing clusters to deal with large amounts of data and computing intensive simulation models. For ensuring reproducibility of modeling results, efficient programming, proper documentation, archiving, and version management are necessary.

Focusing more and more on the computational aspects of modeling entails the danger of neglecting interpretation and synthesis on a conceptual level. Here lies an important task for strategic models, such as systems dynamics models [33], that describe interaction among components of a system more qualitatively than quantitatively. For the field of evolutionary biology, Servedio and colleague, biologists, discussed so-called proof-of-concept models and their value for developing and testing hypotheses [40]. These models are not yet sufficiently established and used, even if their contribution to integrating knowledge over various sectors and disciplines could potentially be large. They have the strong advantage that qualitative results can often be obtained by mathematical analysis, leading to broader and more generalizable insights than computational results.

Well-known examples include the importance of sufficient vaccination coverage for achieving herd immunity, and the increase of average age at infection in vaccinated populations. That these are not merely theoretical results, but may have considerable public health impact, is demonstrated by a recent modeling study. Its authors calculated the increased risk for severe disease outcomes due to the increased age at infection for unvaccinated individuals in highly vaccinated populations, an effect that is often overlooked by vaccine critics, who weigh benefits against risks of vaccination [41]. In a study investigating the impact of vaccination against pertussis, Aguas et al. used a simple mathematical model to show that increased vaccination coverage can lead to an increase in the incidence of severe pertussis cases [42]. Similarly, for foodborne infections with acquired immunity, Swart et al. [43] showed that decreasing environmental contamination with *campylobacter* might lead to an increase in the number of symptomatic infections individuals experience during their lifetime. The increasing focus on computational models has led researchers of all disciplines and users of modeling studies to underappreciate analytic results and theoretical insights. More generally, using modeling to generate knowledge requires a scientific approach, and a modeling study should lead to new insights on the topic at hand. Computation is only a small part of this process, whereas model design and interpretation are just as important for a good modeling study.

## Embedding into public health

How can we organize expertise in mathematical modeling in a large public health institute to ensure high quality of disease modeling? There are two opposing tendencies, centralization and decentralization, with arguments for and against each.

- A central modeling unit working for the entire institute has the advantage of a critical mass of modelers who can exchange ideas and information about technical aspects of modeling and can act as an internal peer review group to ensure best modeling quality.
- Decentralizing modelers, integrated in working groups with researchers of other fields (biology, epidemiology, immunology, environmental sciences), has the advantage of ensuring communication between modelers and researchers from other disciplines from the start. This improves synergy of diverse research fields, to improve alignment of study design (i.e., data collection) and model development, and generates innovative research ideas. The disadvantage is that modelers may become isolated from other modeling colleagues and get insufficient feedback on technical aspects of their work [44]. Experience shows recurring movement from more centralized to decentralized organization of modeling at large public health institutes (such as those mentioned above).

The situation of mathematical modeling in an institute that generates, compiles, and interprets scientific knowledge for policymakers is similar to that of other methodological disciplines, such as bioinformatics. That is because modelers work on a broad range of topics in interdisciplinary projects and are, therefore, often scattered across a variety of units in their organizations. For the infrastructural organization of bioinformatics, Kallioniemi recommended distinguishing two organizational levels: a central core unit operating institute-wide and smaller peripheral units embedded in research groups [45]. These authors stress the importance of sufficient contact of the embedded units with the others; the core unit can facilitate this. Also for mathematical modeling, an organizational structure that combines advantages of centralization and decentralization is preferable, because it retains the close collaboration of modelers with scientists of other disciplines while also providing sufficient critical mass for methodological feedback for modelers. The central core unit needs to assure transparency of its tasks and responsibilities for the entire institute. Each institute needs to budget funds to organize activities and communication for connecting modelers working outside the core unit to those inside it.

**Fig. 2:**
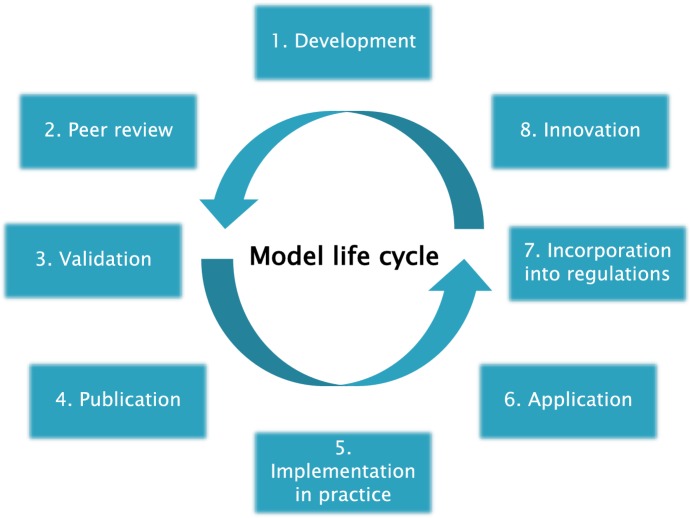
Life cycle of a model. The phases in Fig. 2 are not necessarily occurring in exactly that order in time. Publication can take place at several points in the cycle. The first publication could be on the theoretical framework of the model that can be before validation using real data. Later on, applied analyses using a validated version of the model can also be peer-reviewed and published

Non-modelers usually underestimate how much time and budget is needed to develop and maintain mathematical models. The entire modeling cycle (Fig. 2) requires collaboration and input from many disciplines and stakeholders, but many of the steps of the cycle do not receive sufficient funding in regular institutional budgets often geared to having modelers answer ad hoc questions of policymakers rather than consolidating of results in the context of a long-term strategy. Model development is often performed in projects with limited duration, after which it is difficult to update and maintain the model [44]. Also, elements of implementation are often neglected. Dissemination of results to stakeholders and policymakers often receives less attention, and little allocated time or budget. Consequently, models are often poorly maintained and are not exploited fully for public health policy support [4]. For example, disease models used for generating projections for the national public health ‘foresight studies’ [46] suffer from underfunding and a lack of critical mass of modelers to keep the model up to date with respect to modeling methods and input data [44]. (Foresight studies aim to predict developments in population health and help policymakers to anticipate problems they need to deal with in the future.) For improvement, structural support of modeling activities independent of specific research and policy questions is necessary.

Also, public health institutes and in particular modelers themselves need to make more effort to communicate to policymakers and to the general public what the contributions are of modeling to evidence-based decision-making [4, 22]. Contributions of modeling to evidence-based decisions in public health policymaking are often not explicitly acknowledged, while they are instrumental in generating projections and estimates of health impact of interventions. A prominent example was the decision of the minister of health in the Netherlands about intervention strategies during the 2009 influenza pandemic [47, 48]. Mathematical modelers generated advice, almost daily, about the possible impact of vaccination, about vaccination strategies, and about number of vaccine doses needed. While use and impact of vaccination was discussed nationwide by media and public, most were never aware of the basis on which the Dutch Health Council provided advice and the minister of health decided about vaccine stockpiles and strategies. Thus, the value of the modeling could not be judged by a broader audience [49]. To improve the situation in the future, public health managers could organize communication between modelers and policymakers in so-called communities of practice, (i.e., groups that share a common concern such as a specific public health problem and try to find solutions together interactively), as suggested by Driedger et al. [50].

Finally, to maintain modeling expertise within a public health institute, it is important to have a long-term recruitment strategy with opportunities for young researchers. Institutes also need career options for senior staff [44]. To achieve a balanced composition of staff in terms of age and expertise, strategic planning for the institute should entail a vision for how to develop modeling staff and expertise. To consolidate modeling expertise in the institute, continuous education of staff is necessary, for example, by offering researchers opportunities to participate in conferences and workshops. The central unit can organize training activities to establish common standards for software use and documentation, and these events can increase communication between modelers.

## Modeling research and application

Mathematical modeling for public health is a field that requires scientific thinking and precision, development of novel methods, and a broad perspective to integrate knowledge from other fields. To use modeling potential effectively, mathematical modeling tools need to be ‘state of the art’ in terms of mathematical formulation, implementation into computer code, numerical algorithms, and statistical approaches [51]. Mathematical modeling is a scientific discipline that entails mathematical modelers having the ambition to do research in modeling (including space to do so), publish in dedicated journals, and participate actively in activities of the growing international modeling community. It is essential for a public health institute to have expert modelers who produce and publish internationally recognized modeling studies. The research questions pursued should be inspired by public health policy questions, articulated by public health colleagues with varied expertise and those with whom they interact, along with questions of concern to the general public as voiced by media (discussion about risks and benefits of vaccination). Results should be judged by their applicability for policymakers [22]. This is a delicate balance that requires continuous critical assessment of scientific and societal impact of modeling studies.

Modelers have an important role to play in key issues of modern societies. They are needed for analysis of big data, interpretation of data obtained by data mining, and machine learning. And mathematical models will be needed to assess the impact of e-health and artificial intelligence on public health [5, 52]. Examples of these developments are the use of internet data streams for infectious disease forecasting [53], and more generally, the development of digital epidemiology as a field where collection of digital data, machine learning, and computational science come together [54, 55]. Mathematical modeling is an integral part of these emerging interdisciplinary research fields.

## Conclusions and recommendations

We provide conclusions and recommendations, based on our experience at a national public health institute [23, 44]. We hope these will be useful for other public health institutes or organizations, which use mathematical modeling as a tool for policy support.

- Modeling is more than computation: it is a method of abstraction and understanding complex systems in a systematic manner.
- Modeling needs to be a scientific activity with state of the art approaches, if it is to produce relevant answers to policy questions.
- Contributions of modeling to the evidence base of policy decisions should be acknowledged more explicitly.
- For modeling to be of maximal value for a public health institute, the organization and budgeting of mathematical modeling in the institute should be transparent.
- A long-term strategy for how to position and develop mathematical modeling within the institute and with external partners should be in place.

As public health policy decisions are becoming more complex in a globalized and digitalized world, the benefits that mathematical models can offer for analyzing problems and quantifying the possible impact of interventions are huge [5]. These benefits can only be fully reaped, if mathematical modeling is sufficiently supported and facilitated within the organization of public health institutes.
